# Septic Myocarditis Masquerading as Acute Inferior ST-Elevation Myocardial Infarction

**DOI:** 10.7759/cureus.98607

**Published:** 2025-12-06

**Authors:** Subrata Lahiri, Ovais Rashid, Anshu Goel, Arun Moondhra

**Affiliations:** 1 Department of Cardiology, Sant Parmanand Hospital Civil Lines, New Delhi, IND; 2 Department of Medicine, Pt. Madan Mohan Malaviya Hospital, New Delhi, IND; 3 Department of Internal Medicine, Sant Parmanand Hospital Civil Lines, New Delhi, IND

**Keywords:** acute phase response, cardiovascular disease, myocardial infarction, myocarditis mimicking stemi, pediatric clinical cardiology, sepsis-induced cardiomyopathy, septic shock in children

## Abstract

Acute ST-elevation myocardial infarction (STEMI) is a rare and alarming presentation in adolescents, particularly in women. We report the case of a 16-year-old female who presented with classic symptoms of acute myocardial infarction, including severe retrosternal chest pain, ST-segment elevations on ECG, and a significantly elevated troponin I level (18 ng/mL). The ECG showed evidence of ST elevation in inferior leads, suggestive of inferior STEMI. Urgent coronary angiography revealed normal coronary arteries, effectively ruling out coronary occlusion. The diagnostic pivot was guided by concomitant fever, hypotension, profound leukocytosis (40,900/µL), and a markedly elevated procalcitonin level (11.1 ng/mL). Blood cultures were negative. A transthoracic echocardiogram revealed severe global left ventricular dysfunction (35%). Despite negative blood cultures, a diagnosis of septic shock with secondary septic myocarditis was made based on the overwhelming clinical and biomarker evidence. Initiation of antibiotic therapy led to rapid clinical improvement within 72 hours, accompanied by complete resolution of ST-segment elevations. This case underscores septic myocarditis as a critical mimic of STEMI and highlights the importance of a normal angiogram in redirecting management toward life-threatening infectious etiologies.

## Introduction

The presentation of chest pain with ST-elevations on ECG mandates an immediate rule-out of acute myocardial infarction [1]. However, in young patients without cardiac risk factors, alternative diagnoses must be aggressively pursued. Myocarditis is a well-known impostor, accounting for a significant proportion of ST-elevation myocardial infarction (STEMI) mimics, particularly in adolescent and young adult populations [2]. The diagnostic challenge intensifies when myocardial inflammation occurs secondary to a systemic bacterial infection, a condition termed septic myocarditis. This entity involves acute myocardial dysfunction triggered by a systemic inflammatory cascade, often mimicking STEMI with striking accuracy in both its clinical presentation and electrocardiographic findings [3]. In such cases, the presence of fever, hemodynamic instability, and markedly elevated inflammatory biomarkers becomes a crucial diagnostic red flag, steering the clinical approach away from coronary occlusion and toward a life-threatening infectious etiology [4].

## Case presentation

**Figure 1:**
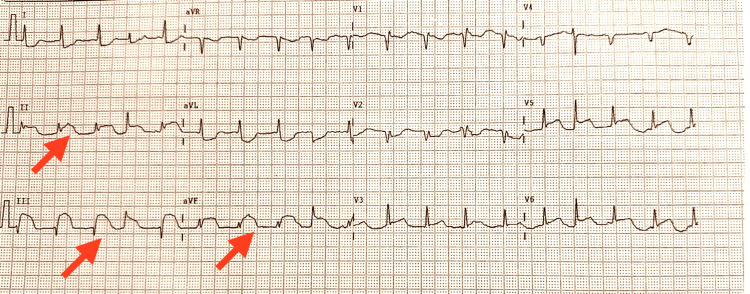
Admission ECG The initial 12-lead ECG shows significant ST-segment elevations in the inferior leads (II, III, and aVF), indicated by red arrows.

A 16-year-old female with no significant past medical history presented to the emergency department following a 24-hour prodrome of high-grade fever and chills, which culminated in the acute onset of crushing substernal chest pain radiating to her jaw. The pain was associated with severe shortness of breath, diaphoresis, and nausea. Upon admission, she was critically ill, with vital signs significant for fever (101.4°F/38.6°C), tachycardia (heart rate 128 bpm), hypotension (70/40 mmHg), tachypnea, and hypoxia. Physical examination revealed a pale, diaphoretic patient in marked respiratory distress. Cardiac auscultation was notable for tachycardia and a soft apical systolic murmur, while pulmonary examination revealed bibasilar crackles. No obvious source of infection was identified on the initial survey. An immediate 12-lead ECG demonstrated significant ST-segment elevations in the inferior leads (II, III, and aVF), as shown in Figure 1.

**Figure 2:**
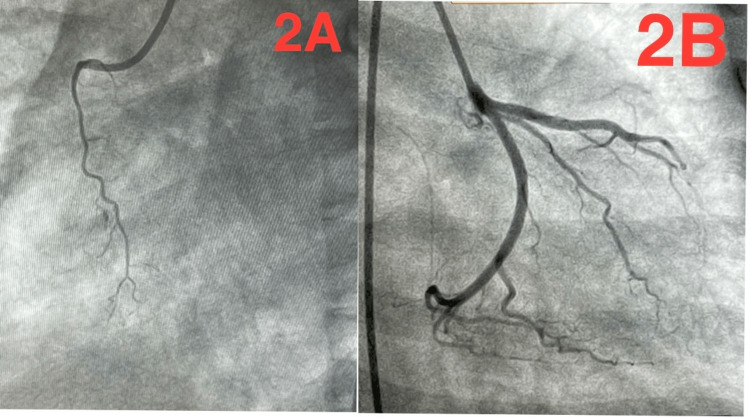
Normal coronary angiogram (A) Right coronary artery in the left anterior oblique projection, non dominant, normal, showing no evidence of occlusion or stenosis. (B) Left coronary system in the right anterior oblique projection with caudal angulation, showing normal left anterior descending and left circumflex arteries.

Point-of-care testing confirmed a significantly elevated troponin I level of 18 ng/mL. In accordance with STEMI protocols, the patient was initiated on antiplatelet therapy and urgently transferred to the cardiac catheterization laboratory. Urgent coronary angiography revealed completely normal coronary arteries, without evidence of thrombosis, dissection, or vasospasm, as shown in Figure 2.

This critical finding definitively excluded acute coronary syndrome and prompted an immediate diagnostic pivot. Subsequent laboratory investigation confirmed a severe systemic infection, with profound leukocytosis (40,900/µL, with neutrophilia) and a normal platelet count (425 × 10⁹/L). Inflammatory markers were markedly elevated, including significantly raised C-reactive protein and a procalcitonin level of 11.1 ng/mL. Arterial blood gas analysis on 80% FiO₂ revealed severe hypoxemia (pO₂ 49.4 mmHg), a markedly elevated lactate level (9.69 mmol/L), and compensatory respiratory alkalosis (pH 7.463). Blood cultures drawn prior to antibiotic administration ultimately returned negative after five days of incubation. Laboratory findings on admission are summarized in Table 1.

**Table 1:** Key laboratory findings on admission

Test	Patient value	Reference range
Troponin I	18 ng/mL	0.010-0.050 ng/mL
Procalcitonin	11.1 ng/mL	0.10-0.50 ng/mL
White blood cell count	40,900/µL	4,000-10,500/µL
Lactate	9.69 mmol/L	<2.0 mmol/L
pH (arterial)	7.463	7.350-7.450
pO₂ (on 80% FiO₂)	49.4 mmHg	80-100 mmHg (on room air)

**Figure 3:**
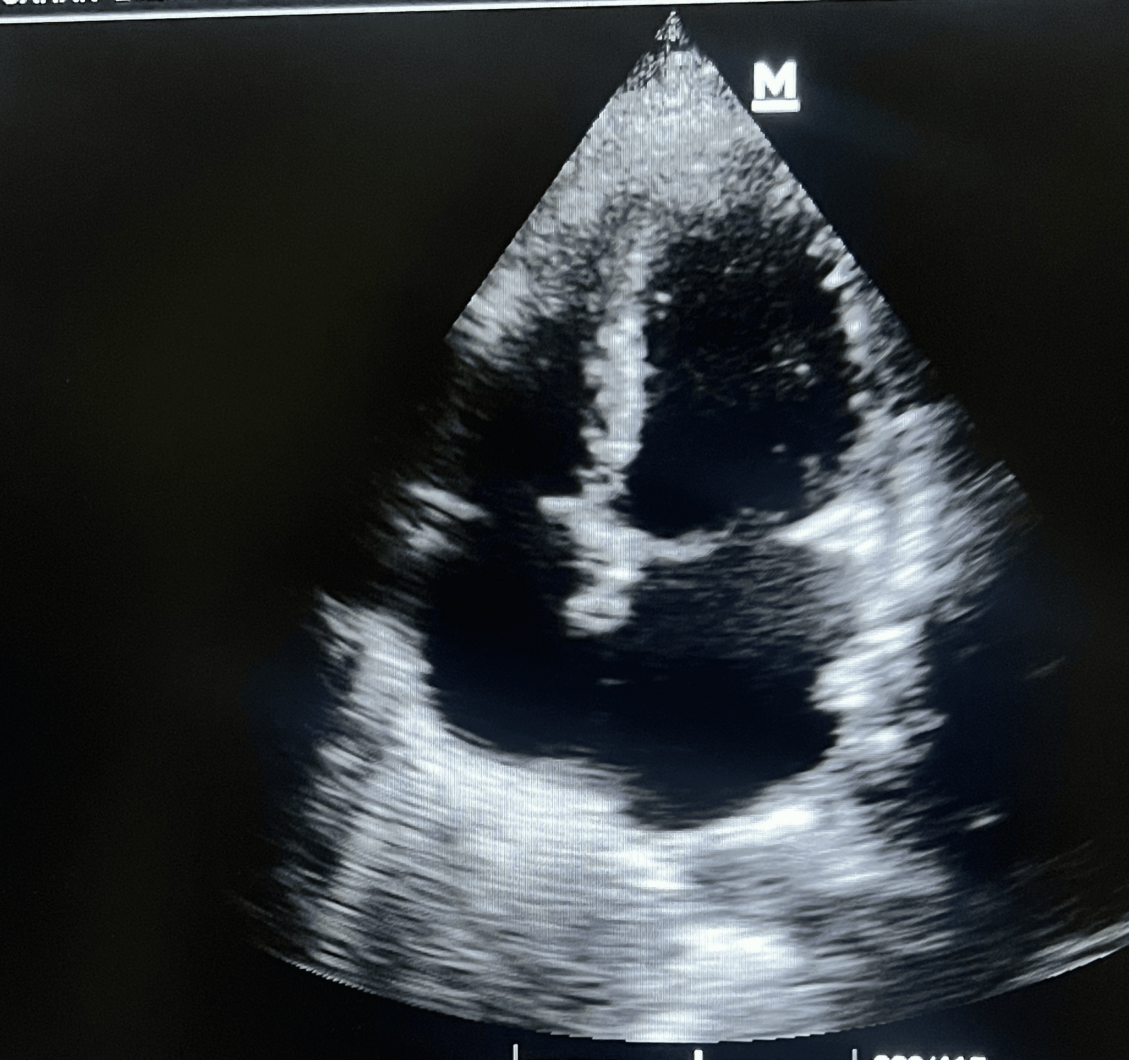
Admission 2D echo The initial 2D echocardiogram shows severe biventricular dysfunction and global hypokinesia.

A comprehensive transthoracic echocardiogram demonstrated severe biventricular systolic dysfunction, with a left ventricular ejection fraction (LVEF) of 35% and global hypokinesia. Additional findings included Grade III left ventricular diastolic dysfunction, moderate mitral regurgitation, mild tricuspid regurgitation, and an estimated right ventricular systolic pressure of 35 mmHg. All cardiac chambers were of normal size, with no evidence of intracardiac thrombus, vegetation, or pericardial effusion (Figure 3).

**Figure 4:**
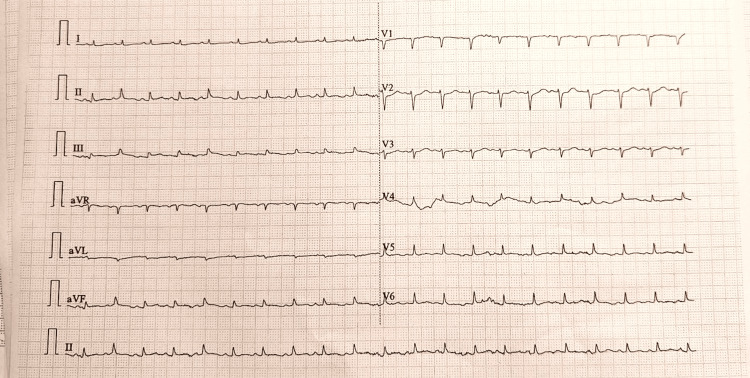
Follow-up ECG A repeat 12-lead ECG shows complete resolution of the previously noted ST-segment elevations in the inferior leads.

The final diagnosis was septic shock from an unidentified source, complicated by severe septic myocarditis and acute cardiomyopathy. All antiplatelet agents were discontinued, and management focused on the underlying septic shock. This included the immediate initiation of empirical broad-spectrum intravenous antibiotics, aggressive fluid resuscitation, and vasopressor support to maintain perfusion, alongside supportive care for acute heart failure and hypoxia. The patient responded dramatically to this targeted therapy. Within 72 hours, she became afebrile and hemodynamically stable, allowing for weaning of vasopressor support. A follow-up ECG confirmed complete resolution of the prior ST-segment elevations (Figure 4).

**Figure 5:**
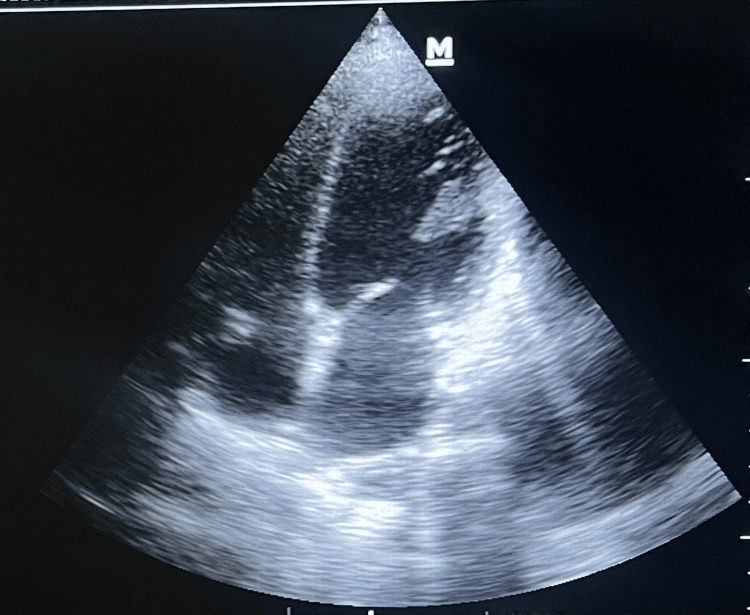
Follow-up 2D echo A repeat 2D echocardiogram shows marked improvement in left ventricular systolic function.

Her leukocytosis, procalcitonin, and troponin levels trended down significantly. A subsequent echocardiogram prior to discharge showed marked improvement in left ventricular systolic function (Figure 5).

The patient was ultimately discharged in stable condition on a complete course of oral antibiotics.

## Discussion

This case is a paradigmatic example of a critical diagnostic dilemma, where a catastrophic systemic infection perfectly mimicked a primary cardiac event. The initial presentation with chest pain, ST-elevations, and troponin rise understandably triggered the STEMI protocol. However, the presence of fever and distributive shock was a crucial red flag pointing toward a systemic illness [4]. The pivotal diagnostic step was the normal coronary angiogram, which definitively excluded coronary occlusion and prevented the unnecessary continuation of dual antiplatelet therapy.

The diagnosis of septic myocarditis was solidified by integrating key findings: the markedly elevated procalcitonin, a highly specific marker for severe bacterial infection [5], leukocytosis, and lactic acidosis. The echocardiogram findings of global hypokinesia and severe systolic dysfunction (LVEF 35%) are more characteristic of diffuse myocardial stunning induced by a cytokine-mediated process in sepsis than a regional coronary event [3]. The rapid resolution of both clinical symptoms and ECG changes with antibiotics alone is pathognomonic for septic myocardial injury rather than permanent infarction.

The fact that blood cultures ultimately returned negative does not invalidate the diagnosis of septic shock and septic myocarditis. It is well-documented that a significant proportion of patients with severe clinical sepsis, supported by markedly elevated inflammatory markers such as procalcitonin, have negative blood cultures [6]. Potential reasons include prior administration of antibiotics (though cultures were drawn before initiation in this case), infections localized in tissues that do not readily cause bacteremia, or pathogens that are fastidious or difficult to culture. The clinical picture, such as fever, leukocytosis, profound hypotension, elevated lactate, and, most specifically, the markedly elevated procalcitonin level of 11.1 ng/mL, remains overwhelmingly indicative of a severe bacterial process [5]. The dramatic and rapid response to antibiotic therapy further supports this diagnosis.

This case underscores several critical learning points for clinicians. First, STEMI must be considered a diagnosis of exclusion in the young; in a febrile adolescent with “STEMI,” mimics like myocarditis must be top considerations before committing to antiplatelet therapy. Second, coronary angiography serves a dual purpose: it is both a therapeutic tool for true STEMI and a vital diagnostic tool that, when normal, mandates an immediate search for alternate causes such as sepsis. Third, biomarkers such as procalcitonin are crucial discriminators, as their significant elevation strongly differentiates bacterial sepsis from other causes of myocardial injury. Fourth, a rapid clinical and electrocardiographic response to antimicrobial therapy can itself be diagnostic, effectively confirming an infectious etiology. Finally, this case reinforces that sepsis remains a clinical diagnosis, and negative cultures do not rule it out in the face of overwhelming supportive evidence.

## Conclusions

This case highlights a significant diagnostic pitfall in emergency and cardiac care. Septic myocarditis can mimic acute STEMI with remarkable fidelity. A normal coronary angiogram should serve as an immediate catalyst to investigate and treat underlying septic shock. Recognizing this entity is crucial, as its management, such as aggressive antibiotic therapy and supportive care, differs completely from that of acute coronary syndrome and is essential for a successful outcome. Maintaining a high index of suspicion for this mimic in young, febrile patients is paramount.
